# Impacts of Calorie Restriction and Intermittent Fasting on Health and Diseases: Current Trends

**DOI:** 10.3390/nu12102948

**Published:** 2020-09-25

**Authors:** Hae Young Chung, Dae Hyun Kim, EunJin Bang, Byung Pal Yu

**Affiliations:** 1 Department of Pharmacy, College of Pharmacy, Pusan National University, 2, Busandaehak-ro 63 beon-gil, Geumjeong-gu, Busan 46241, Korea; bioimmune@hanmail.net (D.H.K.); eunjin2285@gmail.com (E.B.); 2 Department of Physiology, The University of Texas Health Science Center at San Antonio, San Antonio, TX 78229, USA; yu6936@sbcglobal.net

This special issue on the effects of calorie restriction (CR) and intermittent fasting (IF) on health and diseases includes five scholarly reviews and four original articles that provide an insight into the molecular and cellular action mechanisms of epigenetically manipulated dietary paradigms.

CR and IF are well publicized methods for health promotion and disease prevention. An interesting aspect shared by both is that their efficacy is demonstrable without modification of any dietary ingredient. CR, also often called as restriction in dietary intake, provides dietary allocation (generally 10–50% of ad libitum feeding) to the experimental group [1]. Currently, gerontologists have acknowledged CR as the only well-founded anti-aging experimental model and the gold standard in investigating aging interventions. CR intervention delays biological aging and attenuates many age-related pathological processes, thus extending both average and maximal lifespan. Notably, CR is well-known to modulate adulthood diseases, such as atherosclerosis, diabetes, obesity, cancer, and other metabolically related disorders [2].

One unique feature of CR, first proposed as growth retardation in 1935 [3], is its well-known effect on various species, such as rats, mice, hamsters, dogs, yeast, fish, flies, monkeys, and humans [4]. Although many hypotheses for CR diverse actions have been proposed previously, at present, the precise underlying mechanisms remain to be proven. One attractive and plausible mechanism is its highly effective anti-oxidative action against oxidative stress-induced aging. The anti-aging effects of CR are likely attributable to its ability to prevent ubiquitous redox stress by maintaining a homeostatic cellular oxidative status and an antioxidative defense system that contributes to appropriate cellular signaling transductions and proper gene transcription activity [5]. One of the oxidative stress-related physiological processes is the activation of inflammation. The key pro-inflammatory molecules, IL-1β, IL-6, TNFα, iNOS, and COX-2 are all alleviated by CR during aging [6]. CR implementation leads to epigenetic alterations, such as histone modifications and methylation of promoter CpG islands, resulting in epigenetically modified markers in histones and chromatin. This established a molecular mechanism for gene transcription and expression regulation [7,8]. In this regard, lymphocyte-specific protein tyrosine kinase as a new target molecule of the CR modulatory effect was found by integrative analysis using cDNA microarray and interactome [9].

Recent progress in Omics technologies has contributed to technical improvement that allows analysis at the level of the RNA, DNA, protein, and other cellular molecules and their intricate associations during aging. Therefore, it is now feasible to both resolve and coordinate complex biological processes during aging using recent methods, such as next-generation sequencing, proteomics, lipidomics, metabolomics, and epigenomics techniques. Systems biology and Omics integration can predict CR effects, cellular signaling pathways, and action mechanisms. These technical advances made in recent years provide a much-needed database on the regulation of inflammatory process. An expanded view of the inflammatory response in aging progression dubbed as “senoinflammation,” which describes both chronic inflammation and metabolic dysregulation, was suggested in our previous work [10]. Based on previous studies, we observed that the senescence-associated secretory phenotype (SASP) comprises cytokines and chemokines, and these were notably upregulated during aging. This senoinflammatory response was reversed and downregulated by CR [11]. Moreover, metabolic signaling pathways at the cell level were also dysregulated in aging progression, and CR mimetics could reverse such effects. According to CR and Omics big data analysis, cytokines and chemokine pathways are upregulated during aging and modulated by CR [12]. CR is widely accepted as a positive and reasonable control for anti-aging intervention, which recovers disturbed metabolic pathways and decreases the pro-inflammatory SASP [11].

IF is a dietary pattern alternating between fasting and non-fasting periods. Specifically, the IF diet in a particular mouse strain extended both mean and maximal lifespans [13]. For example, loss of body weight and extension of maximal lifespan were observed in 2-, 6-, and 10-month-old C57BL/6J mice with IF implementation [14]. Furthermore, IF lowered the occurrence of diabetes and levels of fasting glucose and insulin [15]. These effects of IF are similar to those observed with CR. The beneficial results of IF on different cancers are also explained by many research groups [16]. The observations in animals indicate that, owing to dietary intake reduction, IF could effectively regulate the number of risk factors and thereby prevent chronic diseases. Such modulatory effects of IF are similar to those of CR. Although the effectiveness of IF has been reported primarily based on in vivo models, implementation of IF in humans has been proposed to prevent major risk factors for age-associated diseases [17]. Systems gerontology (i.e., the study on the overall mechanisms of complex aging progression as an integrated network of systemic interacting molecular pathways, organs, and components during aging) based on Omics technology and systems biology should facilitate unraveling of mechanisms underlying the anti-aging actions of CR and IF as well as their similarities and dissimilarities for better dietary strategies.

The growth hormone (GF)/insulin-like growth factor-1 (IGF-1) axis is an evolutionary well-conserved signaling axis, and the mechanisms underlying its effects on aging and longevity are modulated by CR in mammals [18]. Recently, Shimokawa’s group confirmed that neuropeptide Y (Npy) and FoxO1, which are targets of CR, play various roles in aging and age-associated diseases depending on the nutritional intake [19]. The longevity effects of CR and suppression of IGF-1 signaling pathway are sexually heterogeneous. The beneficial effects of Npy during CR and the diverse roles for Npy in life stages are also described. These genes that mediate the effects of CR and control the aging process depend on nutritional states [19]. As described in a review by Higami’s group, the mitochondrial biogenesis, following CR implementation, in white adipose tissue is regulated by peroxisome proliferator-activated receptor γ coactivator-1α (PGC-1α), a transcription cofactor regulated by sterol regulatory element-binding protein 1c (SREBP-1c), which is the principal regulator of fatty acid biosynthesis [20]. This research group has also suggested that CR mediates the increase in SREBP-1 and PGC-1α, which could occur because of downregulation of the leptin signaling axis and upregulation of fibroblast growth factor 21 (FGF21) expression in white adipose tissue [21].

Another cellular activity recently that was revealed as one of the major players in CR action is the involvement of autophagy. Autophagy is an important cellular management process that maintains an optimal cellular homeostatic condition under normal physiological and abnormal pathological states. Recent developments in CR research have proposed that the initiation of autophagy is associated with beneficial anti-aging response. CR-promoted autophagy plays a critical role under physiological states by sustaining proper homeostasis in the organism. In addition, autophagy induced by CR plays a protective role in different organs and tissues under many pathological and aging-associated conditions [22]. While CR is the most beneficial intervention to prolong lifespan and prevent aging-related diseases, its positive effects on skin aging and diseases are not yet fully understood. CR promotes anti-inflammatory and anti-oxidative responses, stem cell maintenance, and metabolic activities, and such effects contribute to the positive effects on skin aging [23]. Furthermore, widely known CR mimetics, for example, resveratrol, metformin, rapamycin, and peroxisome proliferator-activated receptor agonists, exhibit CR-like effects to inhibit or delay skin aging [23].

Fasting is known to account for physiological changes in the endocrine organs, particularly the pancreas. The study on every other day IF implementation for up to 3 months in a developing and matured healthy organism suggested that this regimen promotes β-cell dysfunction and muscle mass loss and increases fat reserves, especially in developing 30-day-old Wistar rats. More long-term studies are needed to explain the most effective IF regimen to minimize side effects [24]. In recent years, fasting-like intervention methods, such as IF and time-restricted feeding, have appeared as alternatives to CR. Lifespan responses in both CR and IF also differ significantly between males and females [25,26], leading to additional complications in explaining the molecular mechanisms of CR. A simple explanation of these in vivo studies is that a particular regimen of CR and IF may not be effective; however, they may be rather harmful possibly owing to genetic variations and sex [27]. Therefore, for implementation and practice of CR and IF in humans, it is suggested that personalized genomics and medicine should first be set in place to make the most of CR and IF [28].

In summary, we believe that this special issue provides useful information on the current research trends regarding the beneficial effects of CR and IF on longevity and health of humans. Further exploration of the effect of CR and IF on aging and age-related diseases will provide deeper insights into the interrelationships between health and diseases and enable beneficial interventions in aging mechanisms, thus aiding the development of new therapeutic approaches to improve health, diseases, and longevity.

## References

[1] Simpson S.J., Le Couteur D.G., Raubenheimer D., Solon-Biet S.M., Cooney G.J., Cogger V.C., Fontana L. (2017). Dietary protein, aging and nutritional geometry. Ageing Res. Rev..

[2] Devarajan A., Rajasekaran N.S., Valburg C., Ganapathy E., Bindra S., Freije W.A. (2019). Maternal perinatal calorie restriction temporally regulates the hepatic autophagy and redox status in male rat. Free Radic. Biol. Med..

[3] McCay C.M., Crowell M.F., Maynard L.A. (1935). The effect of retarded growth upon the length of life span and upon the ultimate body size. J. Nutr..

[4] Bordone L., Guarente L. (2005). Calorie restriction, SIRT1 and metabolism: Understanding longevity. Nat. Rev. Mol. Cell Biol..

[5] Yu B.P., Chung H.Y. (2006). The inflammatory process in aging. Rev. Clin. Gerontol..

[6] Kim D.H., Kim J.Y., Yu B.P., Chung H.Y. (2008). The activation of NF-κB through Aktinduced FoxO1 phosphorylation during aging and its modulation by calorie restriction. Biogerontology.

[7] Kim C.H., Lee E.K., Choi Y.J., An H.J., Jeong H.O., Park D., Kim B.C., Yu B.P., Bhak J., Chung H.Y. (2016). Short-term calorie restriction ameliorates genomewide, age-related alterations in DNA methylation. Aging Cell.

[8] Stenvinkel P., Karimi M., Johansson S., Axelsson J., Suliman M., Lindholm B., Heimbürger O., Barany P., Alvestrand A., Nordfors L. (2007). Impact of inflammation on epigenetic DNA methylation—A novel risk factor for cardiovascular disease?. J. Intern. Med..

[9] Park D., Lee E.K., Jang E.J., Jeong H.O., Kim B.C., Ha Y.M., Hong S.E., Yu B.P., Chung H.Y. (2013). Identification of the dichotomous role of age-related LCK in calorie restriction revealed by integrative analysis of cDNA microarray and interactome. Age.

[10] Chung H.Y., Kim D.H., Lee E.K., Chung K.W., Chung S., Lee B., Seo A.Y., Chung J.H., Jung Y.S., Im E. (2019). Redefining Chronic Inflammation in Aging and Age-Related Diseases: Proposal of the Senoinflammation Concept. Aging Dis..

[11] Kim D.H., Bang E., Arulkumar R., Ha S., Chung K.W., Park M.H., Choi Y.J., Yu B.P., Chung H.Y. (2020). Senoinflammation: A major mediator underlying age-related metabolic dysregulation. Exp. Gerontol..

[12] Bang E., Lee B., Noh S.G., Kim D.H., Jung H.J., Ha S., Yu B.P., Chung H.Y. (2019). Modulation of senoinflammation by calorie restriction based on biochemical and Omics big data analysis. BMB Rep..

[13] Chung K.W., Kim D.H., Park M.H., Choi Y.J., Kim N.D., Lee J., Yu B.P., Chung H.Y. (2013). Recent advances in calorie restriction research on aging. Exp. Gerontol..

[14] Goodrick C.L., Ingram D.K., Reynolds M.A., Freeman J.R., Cider N. (1990). Effects of intermittent feeding upon body weight and lifespan in inbred mice: Interaction of genotype and age. Mech. Ageing Dev..

[15] Hsieh E.A., Chai C.M., Hellerstein M.K. (2005). Effects of caloric restriction on cell proliferation in several tissues in mice: Role of intermittent feeding. Am. J. Physiol. Endocrinol. Metab..

[16] Descamps O., Riondel J., Ducros V., Roussel A.M. (2005). Mitochondrial production of reactive oxygen species and incidence of age-associated lymphoma in OF1 mice: Effect of alternate-day fasting. Mech. Ageing Dev..

[17] Varady K.A., Hellerstein M.K. (2007). Alternate-day fasting and chronic disease prevention: A review of human and animal trials1’2’3. Am. J. Clin. Nutr..

[18] Chiba T., Yamaza H., Shimokawa I. (2007). Role of insulin and growth hormone/insulin-like growth factor-I signaling in lifespan extension: Rodent longevity models for studying aging and calorie restriction. Curr. Genom..

[19] Komatsu T., Park S., Hayashi H., Mori R., Yamaza H., Shimokawa I. (2019). Mechanisms of Calorie Restriction: A Review of Genes Required for the Life-Extending and Tumor-Inhibiting Effects of Calorie Restriction. Nutrients.

[20] Fujii N., Narita T., Okita N., Kobayashi M., Furuta Y., Chujo Y., Sakai M., Yamada A., Takeda K., Konishi T. (2017). Sterol regulatory element-binding protein-1c orchestrates metabolic remodeling of white adipose tissue by caloric restriction. Aging Cell.

[21] Kobayashi M., Uta S., Otsubo M., Deguchi Y., Tagawa R., Mizunoe Y., Nakagawa Y., Shimano H., Higami Y. (2020). Srebp-1c/Fgf21/Pgc-1α Axis Regulated by Leptin Signaling in Adipocytes-Possible Mechanism of Caloric Restriction-Associated Metabolic Remodeling of White Adipose Tissue. Nutrients.

[22] Chung K.W., Chung H.Y. (2019). The Effects of Calorie Restriction on Autophagy: Role on Aging Intervention. Nutrients.

[23] Choi Y.J. (2020). Shedding Light on the Effects of Calorie Restriction and its Mimetics on Skin Biology. Nutrients.

[24] Munhoz A.C., Vilas-Boas E.A., Panveloski-Costa A.C., Leite J.S.M., Lucena C.F., Riva P., Emilio H., Carpinelli A.R. (2020). Intermittent Fasting for Twelve Weeks Leads to Increases in Fat Mass and Hyperinsulinemia in Young Female Wistar Rats. Nutrients.

[25] Liao C.Y., Rikke B.A., Johnson T.E., Diaz V., Nelson J.F. (2010). Genetic variation in the murine lifespan response to dietary restriction: From life extension to life shortening. Aging Cell.

[26] Wilson K., Beck J., Nelson C., Hilsabeck T., Promislow D., Brem R., Kapahi P. (2020). GWAS for Lifespan and Decline in Climbing Ability in Flies upon Dietary Restriction Reveal decima as a Mediator of Insulin-like Peptide Production. Curr. Biol..

[27] Di Francesco A., Di Germanio C., Bernier M., de Cabo R. (2018). A time to fast. Science.

[28] Hwangbo D.S., Lee H.Y., Abozaid L.S., Min K.J. (2020). Mechanisms of Lifespan Regulation by Calorie Restriction and Intermittent Fasting in Model Organisms. Nutrients.

